# Iron Dysregulation During Severe COVID-19: Longitudinal Evidence from Hospitalized Patients

**DOI:** 10.3390/nu18213570

**Published:** 2026-10-29

**Authors:** Elisa Antinori, Alice Vianello, Lorenzo Bertolone, Camila Dos Santos Lima, Fabiana Busti, Misha Fatima, Acaynne Lira Zidanes, Patrizia Pattini, Rossella Gottardo, Elisa Danese, Fabio Chesini, Simonetta Friso, Domenico Girelli, Annalisa Castagna

**Affiliations:** 1 Section of Internal Medicine, Department of Medicine, University of Verona and Azienda Ospedaliera Universitaria Integrata di Verona, 37134 Verona, Italy; 2 Urgency Medicine Unit, Department of Medicine, University of Verona, 37134 Verona, Italy; 3 Unit of Internal Medicine, Department of Medicine, University of Verona, 37134 Verona, Italy; 4 Unit of Forensic Medicine, Department of Diagnostics and Public Health, University of Verona, 37134 Verona, Italy; 5 Section of Clinical Biochemistry, Department of Engineering for Innovation Medicine, University of Verona, 37134 Verona, Italy

**Keywords:** iron metabolism, COVID-19, SARS-CoV-2 infection, hepcidin, inflammation, cytokines

## Abstract

Background/Objectives: The COVID-19 pandemic underlined the critical role of iron homeostasis in infections, particularly in those cases where hyperinflammation, fueled by massive elevation in cytokines such as IL-6, TNF-α, and IL-8, triggered acute respiratory distress syndrome (ARDS), progressive multiorgan failure and heightened mortality. This extreme immune response aggravates systemic iron imbalance and eventually triggers a vicious cycle in which the inflammatory state fuels the impaired iron metabolism, thereby contributing to disease severity. The main goal of this work was to disentangle the relationships between inflammation and iron metabolism in a cohort of well-characterized patients hospitalized for severe COVID-19. Methods: This study evaluated 389 patients hospitalized with COVID-19 pneumonia in a medical ward at Verona University Hospital. Complete clinical parameters, inflammatory, and iron-related markers’ trends were analyzed over time during hospitalization (admission (T0), 1 week (T1), and 2 weeks after (T2)). Subjects were grouped by disease severity during hospitalization. Results: High-severity cases showed significantly higher concentrations of IL-8, IL-6, IL-10, hepcidin, and ferritin at baseline. Hepcidin correlated strongly with inflammatory markers and was a predictor of COVID-19 severity (*p* = 0.014). Despite a gradual decrease, hepcidin levels remained significantly higher in patients with a more serious form of the disease across time points. Conclusions: Our findings underscore the clinical utility of iron-related markers in COVID-19 management and the relevance of their assessment in clinical settings for better patient stratification.

## 1. Introduction

Iron is a fundamental element for almost all living organisms, including humans [1]. Due to its capacity to alternate between oxidation states via electron exchange, iron plays an indispensable role in fundamental mechanisms, such as oxygen transport and host defense. Most of the body iron (80%) is contained in red blood cells (RBCs), where it is necessary for hemoglobin (Hb) synthesis. As there is no physiological means for excreting iron, its amount must be tightly balanced to avoid both excess and deficiency. In fact, long-lasting alterations from the normal plasmatic iron levels can have detrimental effects, such as iron-deficiency anemia or tissue damage from iron overload [2]. Thus, tight control at both the cellular and systemic levels is reached through regulatory mechanisms and key molecular players in the iron cycle, which coordinate iron absorption, transport, and recycling. The fulcrum of this regulation is the ferroportin (FPN)-hepcidin axis: in iron replete condition, the liver-derived hormone hepcidin acts as a negative regulator, binding to FPN, the only iron exporter found in mammals, and inducing its lysosomal degradation [3]. Once released by FPN, iron is transported throughout the body bound to transferrin (Tf), which delivers Fe^3+^ to various tissues, including the bone marrow, where it is used for erythropoiesis. Cellular uptake of iron occurs through the binding of Tf with transferrin receptor 1 (TfR1); its soluble form, known as sTfR, is a valuable biomarker for assessing iron status, particularly in conditions such as iron-deficiency anemia [4].

Approximately 20–25 mg of iron is recycled daily by macrophages, which phagocytize senescent or damaged red blood cells and retrieve the iron from heme; this iron is then released through FPN for reuse in erythropoiesis, a process controlled by hepcidin. This tight regulation is critical in conditions such as hypoxia or severe blood loss, when erythroferrone (ERFE), a recently discovered erythroid regulator responsive to erythropoietin, suppresses hepcidin expression to sustain intensified erythropoiesis [2]. Conversely, during infection and systemic inflammation, iron metabolism is altered to restrict the availability of iron to invading pathogens. In this context, the iron cage Ferritin (FT) acts not only as a storage protein but also as a marker of inflammation and iron status: its synthesis is modulated by iron availability, but also by inflammatory signals such as interleukin-6 (IL-6) [5]. IL-6 and other cytokines also mediate the enhanced production of hepcidin, which reduces iron levels in the bloodstream and thereby limits the growth of extracellular microbes that depend on NTBI (Non-Transferrin Bound Iron) [6]. However, the resulting hypoferremia reduces iron availability for erythropoiesis, thereby contributing to the onset of anemia of inflammation [7,8].

In light of increasing evidence underscoring the interplay between iron status and immunity, biomarkers of iron metabolism have been examined in various pathological conditions, including Coronavirus disease 2019 (COVID-19) [9,10].

COVID-19, due to Severe Acute Respiratory Coronavirus 2 (SARS-CoV-2), was declared a pandemic on 11 March 2020, and has generated more than 7.1 million deaths to date, worldwide. COVID-19 patients (~80%) are mostly asymptomatic or present symptoms such as dry cough, pirexia, and fatigue, which are typical of mild or moderate illness. Viral pneumonia develops in approximately 15% of cases, necessitating hospitalization. Approximately 5% of cases progress to critical illness, characterized by acute respiratory distress syndrome (ARDS), multiple organ failure (MOF) or shock, and need mechanical ventilation or intensive care unit (ICU) admission [9,11,12,13].

COVID-19 patients experience a condition in which two stimuli, that normally control iron metabolism in opposite ways, coexist: on the one hand, high levels of inflammation induce an increase in acute-phase proteins and restrict iron supply through the production of hepcidin and ferritin; on the other hand, hypoxic conditions require iron mobilization to support erythropoiesis by suppressing hepcidin expression.

Given the function of iron in infection and immunity and the complex pathophysiological mechanisms involved in COVID-19, our study aimed at elucidating the relationships and correlations between inflammatory molecules and iron homeostasis in a well-characterized set of patients hospitalized with severe COVID-19. We focused on elucidating the function of classical and emergent iron parameters (hepcidin, ERFE, sTfR, cytokines), collected at three time points, as predictors of disease severity.

## 2. Materials and Methods

### 2.1. Subjects Population

We studied 389 consecutive patients with a diagnosis of SARS-CoV-2 pneumonia who were referred to the Azienda Ospedaliera Universitaria Integrata of Verona, Italy, from April 2020 to May 2021. Data, including demographics, medical history, biochemical parameters, and arterial blood gas tests, were obtained and recorded on a dedicated COVID-19 database. Information on the in-hospital clinical course was retrospectively collected from patients’ medical records. In addition, biological samples for research purposes were collected at 3 time points: at admission (T0), and 1 week (T1) and 2 weeks (T2) later during the hospital stay.

Eligibility criteria included a positive SARS-CoV-2 nasopharyngeal swab subsequently confirmed by testing, collection of a T0 blood sample within 48 h of hospital admission, and a diagnosis of COVID-19 pneumonia. The University Hospital of Verona Ethical Committee approved the study (No. 2646CESC). Given the extraordinary circumstances of the pandemic, the Committee waived the requirement for written informed consent for inclusion in non-interventional and observational studies. Oral informed consent documented in the medical records was deemed sufficient.

### 2.2. Laboratory Analysis

The Central Laboratory of the University Hospital of Verona carried out automatically all the routine tests on blood samples collected by venipuncture.

Ella (ProteinSimple, Bio-Techne, San Jose, CA, USA) an automated instrumentation for multiple immunoassays was used for assessing a panel of cytokines (IL-1 ra, IFNγ, IL-10, IL-1β, IL-22, IL-6, TNF-α and IL-8) on all the available samples. Hepcidin erythroferrone and sTfR measurements were performed by commercially available ELISA kits (Intrinsic LifeSciences, The BioIron Company, La Jolla, CA, USA and DRG Instruments GmbH, Marburg, Germany) according to manufacturer’s instructions. GraphPad Prism 8 software (San Diego, CA, USA) was used for data interpolation and reduction on duplicates. Normal reference ranges and LOD for hepcidin and sTfR were considered according to manufacturers, while for erythroferrone we referred to previously published data [14,15].

### 2.3. Statistical Analysis

Normality was assessed on variables via Shapiro-Wilk test. Continuous variables are reported as mean ± standard deviation (SD) for normally distributed data and as median and interquartile range (25th–75th percentiles) for non-normally distributed data. Categorical variables are presented as counts and percentages.

Student’s *t*-test, when appropriate, or the Mann–Whitney U test otherwise, was used to assess association with disease severity. Categorical variables were compared by the Chi-Squared (χ^2^) test. Spearman’s R correlations analysis was used for evaluating association between variables. Statistically significance was set at two-sided *p* < 0.05. For generating images and statistical descriptives R (4.1.1) or SPSS (IBM SPSS statistics 28) and GraphPad Prism (10.0.1) were exploited.

Changes in the same variables across different time points were analyzed using repeated-measures ANOVA for normally distributed data or the Friedman test for non-normally distributed data. Wilcoxon signed-rank test with Bonferroni correction for multiple comparisons was applied for pairwise post-hoc comparisons. Logistic and linear regressions (multivariate analysis) were obtained by R software.

## 3. Results

The biochemical and hematological profile of the studied population at hospital admission are summarized in Table 1.

Figure S1 shows the population studied subdivided according to disease severity. Stratification criteria included in-hospital mortality and the maximum oxygen requirement according to the definition available in literature for severe COVID-19 [16]. The high severity group included patients who experienced in-hospital mortality or required Orotracheal Intubation (IOT), Continuous Positive Airway Pressure (CPAP), or Non-Invasive Ventilation (NIV). Conversely, subjects requiring either no respiratory support or oxygen delivery only vianasal cannula or simple face mask were classified as having medium/low severity disease.

The patients population included in our study was mainly characterized by male subjects with a median age of 71 years. Severe COVID-19 disease risk increased with male proportion and age. The number of inpatient days between the two severity groups differed in a statistically significant way, with a median of 17 days for the hospitalization for the high-severity group. D-dimer values did not differ significantly between the two severity groups. Immune cell populations peculiar alterations, such as lymphopenia, elevated white blood cell count, neutrophil count, and a high neutrophil/lymphocyte (N/L) ratio were associated to more severe disease progression, as well as higher median CRP levels. A higher RDW was found to be associated with a worse outcome while hemoglobin values and RBC count were within the normal range and didn’t differ in the two groups. In the low/medium severity group the oxygen demand indicator PaO_2_/FiO_2_ (Partial pressure of Oxygen in Arterial blood/Fraction Inspired Oxygen) was significantly higher, as expected. On the other hand, Charlson Comorbidity index (CCI) was not significantly different between the two groups. As for therapy, nearly all patients received steroids and heparin (>90%); remdesivir was administered in 34.5% of cases, and tocilizumab in only 3 patients. Therapy did not reach statistical significance among the two investigated groups. With regard to iron-related parameters (Table 2), the results showed marked hypoferremia and hyperferritinemia. Hepcidin concentrations of the whole population were higher than the normal range; severely ill patients showed hepcidin and ferritin values higher than those of subjects in the low/medium severity group. On the contrary, no significant difference was observed in baseline serum iron, ERFE or transferrin saturation between the two severity groups. ERFE levels in our population were similar to the normal range reported in literature in a study done with the same assay on the same type of biological fluid (0.51 ng/mL (IQR: 0.12–1.25) [14].

Moreover, cytokines profile showed the trends illustrated in Table 3. The high severity group consisted of markedly higher levels of IL-6, IL-8, and IL-10 when compared to the low/medium severity group.

To determine potential predictors of disease severity, a binary logistic regression was performed incorporating the variables that previously showed significant differences between the two severity groups (Table S10). The multivariate model corroborated the univariate findings, identifying hepcidin as a highly significant predictor of disease severity (*p* = 0.004). The model exhibited outstanding discriminating performance, yielding an Area Under the Curve (AUC) of 0.88 (95% CI 0.83–0.93). Furthermore, stratification of hepcidin concentrations into quartiles (Q1: <129.5 ng/mL; Q2: 129.5 ng/mL–200.9 ng/mL, Q3: 200.9 ng/mL–265.3 ng/mL; Q4: >265.3 ng/mL), showed that patients in the highest category (Q4) experienced a four-fold elevation in the risk of developing a more severe form of COVID-19 (Exp(B) = 4) (Table S11 and Figure S2).

To further investigate the possible role of comorbidities and pharmacological therapies, we adjusted the model for CCI and individual drug classes (Table S14). The predominant role of hepcidin remained robust, while neither comorbidities nor specific treatments reached statistical significance.

**Figure 1:**
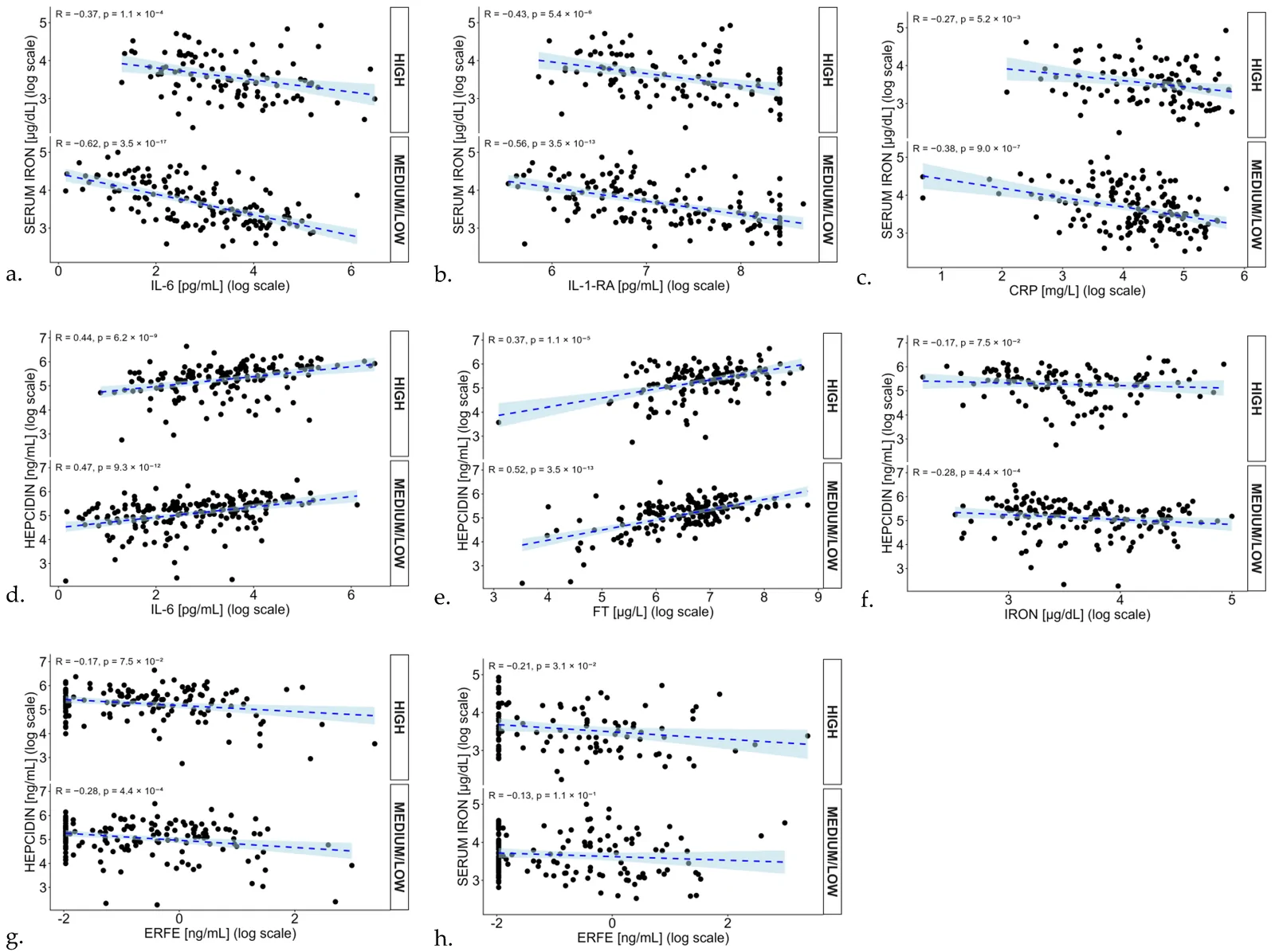
Correlation analysis between inflammatory markers and iron metabolism parameters. Scatter plots show the relationship between parameters: (**a**) Iron vs. IL-6; (**b**) Iron vs. IL-1-RA; (**c**) IRON vs. CRP; (**d**) IL-6 vs. Hepcidin; (**e**) Ferritin vs. Hepcidin; (**f**) Iron vs. Hepcidin; (**g**) Erfe vs. Hepcidin; (**h**) Erfe vs. Iron., stratified by COVID-19 severity (High vs. Medium/Low). All data are presented on a logarithmic scale. The dashed blue lines represent the linear regression fit, with the light blue shaded areas indicating the 95% confidence intervals. Individual patient data are plotted as black dots. Within each panel significance levels (*p*) and Spearman’s correlation coefficients (*R*) are indicated. CRP = C Reactive Protein; FT = Ferritin.

Relationships between most relevant variables were evaluated through spearman correlations (Figure S3). In patients presenting with low to medium severity, serum iron levels demonstrated a negative correlation with inflammatory indicators (IL-6, IL-1-R-A, CRP). Moreover, these correlations was attenuated in the high severity group, suggesting a loss of linear dependence as the condition progresses and immune system dysregulation escalates (Figure 1a–c). Consistent with the aforementioned role in hepcidin induction, IL-6 was positively associated with hepcidin concentrations (rs = 0.46; *p* < 0.001) (Figure 1d). Other inflammatory cytokines showed a similar pattern. Furthermore, an expected robust positive correlation emerged between hepcidin and ferritin levels (rs = 0.46; *p* < 0.001) (Figure 1e). Interestingly, no significant correlation was found between iron and hepcidin in the high severity group (rs = −0.17; *p* = 0.075) (Figure 1f), further confirmed by the multivariate linear regression analysis (Table S12). No correlation was observevd between erythroferrone and hepcidin (rs = −0.12; *p* < 0.001) or erythroferrone and iron (rs = −0.17; *p* < 0.001) (Figure 1g,h).

A multivariate linear regression analysis with hepcidin as the dependent variable was also performed. The variables that showed a statistically significant relationship with hepcidin were IL-1-RA, IL-6, IL-8, CRP, ferritin and N/L (Table S13). In fact, the analysis of incremental explained variance (ΔR^2^) (Figure S4), demonstrates that inflammatory markers provided the largest contribution to the prediction of hepcidin levels (ΔR^2^ = 0.321). Iron availability markers also accounted for a notable portion of the variance (ΔR^2^ = 0.112). In contrast, age and variables related to erythropoiesis and oxygenation showed only marginal incremental effects (ΔR^2^ = 0.005 and 0.040, respectively).

**Figure 2:**
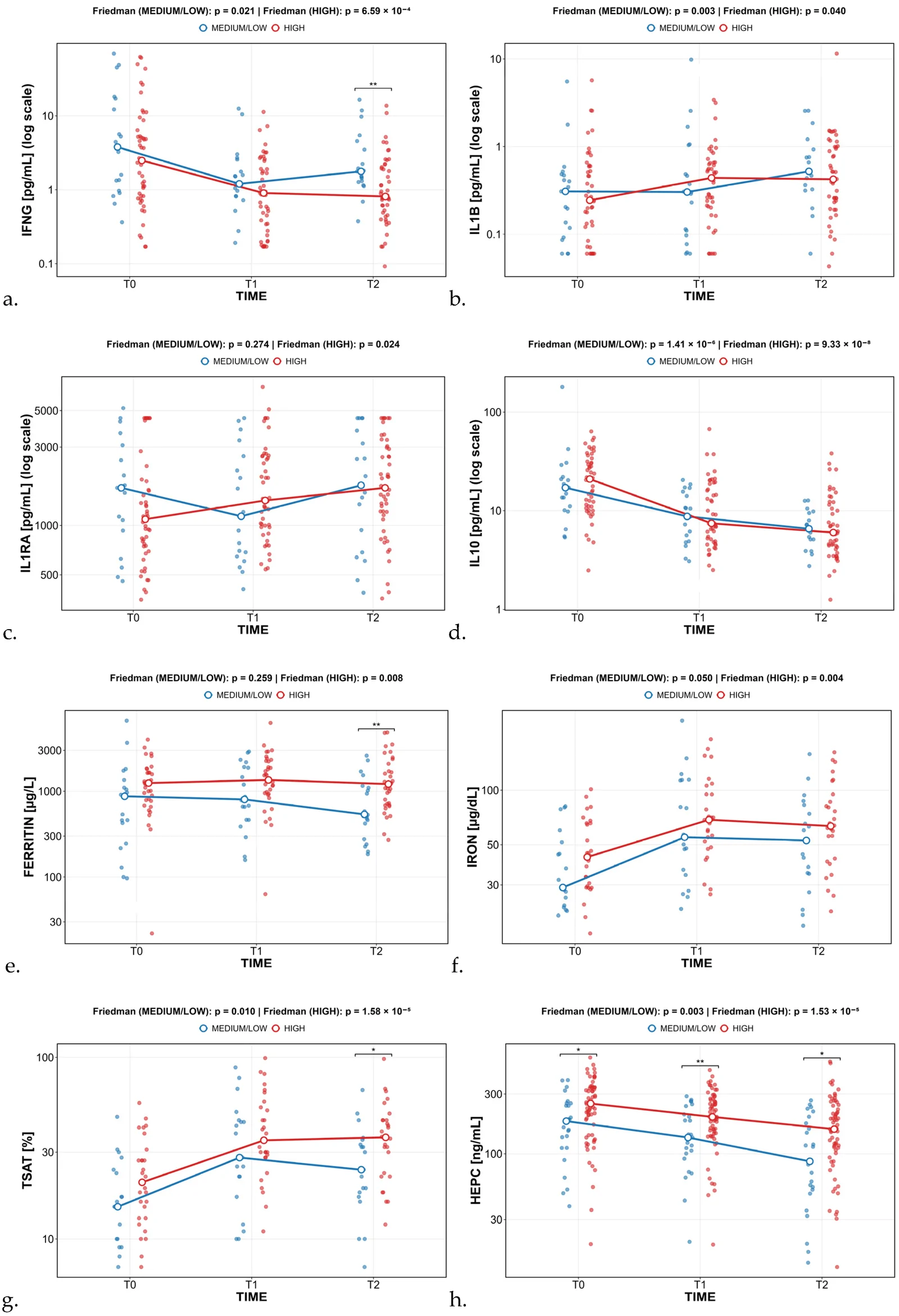
Cytokines, Ferritin, iron, HEPC (hepcidin), TSAT (Transferrin Saturation), behaviour across the different time points. (**a**) IFNγ; (**b**) IL-1β; (**c**) IL-1-RA; (**d**) IL-10; (**e**) Ferritin; (**f**) Iron; (**g**) TSAT; (**h**) Hepcidin. Blue lines represent medium/low severity group. The high severity groups are indicated by red lines. T-bar represents the 75th and 25th percentiles while median values are indicated by dots. * *p* < 0.05, ** *p* < 0.01.

We also investigated clinical and biochemical parameters at T1 and T2 in relation to disease severity (Tables S1–S9). Of note, concerning cytokines, during hospitalization we documented a highly significant and early decrease of IFNγ (3.6 pg/mL at T0, 0.93 pg/mL at T1, 1.15 pg/mL at T2) and IL-10 (16 pg/mL at T0, 7.8 pg/mL at T1, 6 pg/mL at T2) and a delayed increase of IL-1 Ra (1310 pg/mL at T0, 1283.5 pg/mL at T1, 1740 pg/mL at T2) and IL-1β (0.3 pg/mL at T0, 0.4 pg/mL at T1, 0.5 pg/mL at T2) during the 2 weeks period after COVID-19 diagnosis, indicating a progressive decrease of the cytokine storm (Figure 2a–d).

Iron parameters presented peculiar trends as well; ferritin concentrations were significantly higher in the high-severity group after 14 days of hospitalization, presenting a decreasing pattern only in the non-severe group (839 µg/L at T0, 821 µg/L at T1, 537.5 µg/L at T2) (Figure 2e). Serum iron levels in our cohort of patients showed a U-shaped pattern over time, without any significant difference between the two severity groups. However, in the pairwise comparison a significant difference in serum iron levels was obtained only between T0 and T1 (*p* = 0.001), and not between T1 and T2 (*p* = 0.239) (Figure 2f). TSAT, a possible indicator of catalytic iron levels, was significantly different between T0 and T1 (*p* = 0.001), even if not between T1 and T2 (*p* = 0.150) and an U-shaped pattern over time. Moreover, patients with severe COVID-19 had TSAT levels significantly higher compared with patients with other forms of COVID-19, at both T1 and T2 (Figure 2g). In general, hepcidin levels remained high accross the whole hospitalization, though showing a decreasing trend over time (230.4 ng/mL at T0, 188.4 ng/mL at T1, 151 ng/mL at T2) (Figure 2h).

## 4. Discussion

Our retrospective study analyzed, in nearly 400 patients hospitalized with COVID-19 pneumonia, a comprehensive set of cytokines and iron-related markers at three time-points. Rather than focusing on baseline status at admission, we evaluated associations with the worst respiratory status during hospitalization to better capture the overall disease burden. The high and low/medium-severity groups differed in several clinical and biochemical parameters.Consistent with previous literature [17], severe disease was characterized by a neutrophil-predominant rather than lymphocyte-predominant immune response. Lymphopenia may reflect lymphocyte redistribution to affected tissues, cytokine-mediated impairment of lymphopoiesis and survival, and increased activation-induced cell death [18]. Conversely, D-dimer, a well-established marker of fibrinolysis and a predictor of COVID-19 severity [19], did not differ between groups, possibly due to timing of assessment, concomitant anticoagulant therapy, coagulation abnormalities, or impaired kidney function.

The higher RDW in the high-severity group may indicate ineffective erythropoiesis and heterogeneous erythrocyte production under inflammatory stress. Indeed, in a previous study we identified RBC morphological abnormalities in 65% of 115 COVID-19 patients [20], most commonly spiculated cells. Patients with more than 10% abnormal RBCs had more pronounced lymphopenia and thrombocytopenia, supporting an association with sustained immune-inflammatory stress hematopoiesis, and higher mortality.

Given the focus of our study, we specifically examined changes in iron parameters over time and between severity groups. As a general remark and in line with previous studies results [21,22,23,24], our cohort showed marked hypoferremia and hyperferritinemia, a pattern more consistent with inflammation-driven iron sequestration than with increased circulating iron availability. In our study, FT correlated positively with disease severity, consistent with the greater prevalence of persistent hyperferritinemia in severe/critical than in mild cases. Despite being influenced by age, sex, and kidney function, among other factors [25], FT may serve as a disease severity marker and not only as an indicator of iron stores.

In our cohort, patients with severe disease showed significantly elevated sTfR levels, reflecting the increased cellular iron demand and stressed erythropoiesis driven by systemic inflammation and functional iron deficiency. Conversely, ERFE levels did not differ significantly between severity groups. Although ERFE tipically respond to hypoxia, the severe inflammatory environment, characterized by massive IL-6 release and high hepcidin levels, appears to override or decouple the expected compensatory suppression of hepcidin by ERFE.

In our study, hepcidin emerged as the most important finding and the most consistent iron-related parameter associated with disease severity. Notably, it was the sole measured variable to consistently maintain significantly higher levels (exceeding the normal range) in the high-severity group across all evaluation time points, despite a progressive longitudinal decline from 200.88 ng/mL at T0 to 156.87 ng/mL at T1 and 149.98 ng/mL at T2 (Friedman test, *p* < 0.001). While these findings align with studies linking increased hepcidin to severe pneumonia trajectories [26,27,28], published results remain inconsistent, with some reports describing lower or comparable hepcidin levels in critically ill patients [29].

In the more severe group, hepcidin correlated strongly with IL-6 but not with serum iron, mirroring the findings of Moreira et al. [27]. This dissociation may reflect the complex inflammatory and metabolic response to acute infection. In COVID-19 pneumonia, pro-inflammatory cytokines can drive hepcidin production independently of iron status, disrupting the usual feedback mechanism. Multivariate analysis further underlined the dependence of hepcidin on inflammatory markers and immune cells, rather than responding to gender or age variations, suggesting that the uncontrolled inflammatory response overrides physiological stimuli. Inflammatory markers accounted for approximately 21% of the variance in hepcidin, confirming that inflammation is a major driver of hepcidin upregulation in hospitalized patients.

The severity-prediction model identified the fourth hepcidin quartile (>265.3 ng/mL) as particularly informative: a fourfold higher risk of severe COVID-19 was observed among patients in this quartile, confirming the progressive association between higher hepcidin and worse outcomes. Notably, this significance remained robust after adjusting for covariates such as CCI and treatment regimens (including anticoagulants, antivirals, and steroids).

As a general consideration, these results suggest that hepcidin measurement may facilitate early identification of patients at risk of critical disease, although concentrations obtained with different methods should be compared with caution.

Regarding the cytokine panel, patients with severe disease had higher IL-6 levels, consistent with exploratory studies of complicated COVID-19 [30], and higher IL-8 levels, a driver of neutrophil chemotaxis, and a proposed prognostic biomarker [31]. The significant elevation of IL-10 (13.2 pg/mL), a feature of the cytokine storm, may represent a compensatory anti-inflammatory response; however, sustained IL-10 signaling may also contribute to COVID-19 immunopathology and severity [32].

Our findings support a model in which inflammation-driven iron sequestration predominates compensatory mechanisms normally activated by hypoxia (Figure S5). The longitudinal persistence of elevated hepcidin suggests that iron dysregulation accompanies sustained inflammatory activity rather than representing an isolated alteration at admission. Severe infection induces massive IL-6 release, upregulating hepatic hepcidin synthesis. Hepcidin binds to ferroportin, promoting its internalization and degradation, thereby limiting iron release from macrophages and reticuloendothelial cells, causing functional iron deficiency with elevated ferritin. Liver and kidney dysfunction typical of this setting may further alter the hepcidin–iron relationship [33,34]. Under physiological conditions, hypoxia stimulates ERFE-mediated suppression of hepcidin to mobilize iron; however, in severe COVID-19, the inflammatory stimulus overrides this axis, maintaining high hepcidin levels despite suppressed serum iron and increased erythropoietic demand. Elevated sTfR levels alongside unchanged ERFE levels, found in our study, may reflect this imbalance between iron-restrictive inflammatory signaling and compensatory erythropoiesis. The concurrent presence of multiple, even conflicting, mechanisms in severe COVID-19 disease and the complexity of their interaction may also explain our observation related to the weakening of correlations between iron-related and inflammatory markers in the high-severity group.

Nevertheless, we acknowledge that our study has some limitations. Its design as retrospective, single-center study, precludes causal inference and might have included selection bias and residual confounding. Both the enrollment period and evolving standards of care may have influenced the observed associations. The absence of a control group (healthy subjects or patients with other types of pneumonia) limits the assessment of whether the alterations were specific to COVID-19; however, our primary aim was to characterize the longitudinal variations and the prognostic value of iron homeostasis in a cohort of hospitalized COVID-19 patients, rather than perform a comparative analysis between COVID-19 and other infectious diseases or healthy individuals. Moreover, extensive literature evidence has already established that hypoferremia and hyperferritinemia are hallmarks of “anemia of inflammation”, a condition common to various severe infections. Our findings build upon this by detailing how these dynamics specifically unfold and predict trajectories in severe COVID-19 pneumonia over a two-week hospital stay.

Another limitation regards the heterogeneity of our high-severity group which combines CPAP recipients with mechanically ventilated and deceased patients or those that underwent orotracheal intubation. We must consider that in exceptional circumstances, such as during pandemic, resource allocation imposed operational restrictions in internal medicine wards, where some severe patients were managed exclusively with non-invasive support. Despite these operational limitations, those individuals shared a comparable biological and inflammatory burden.

Missing data, lack of some anthropometric parameters (BMI) and variation in the number of patients assessed at each time point may have introduced additional bias, particularly survivor and attrition bias at T2.

## 5. Conclusions

In conclusion, our findings support the prognostic value of iron-related biomarkers in COVID-19 and their potential utility for patient stratification and treatment decisions. Our data contributes to disentangling the complex interrelation of inflammation and iron dysregulation in a cohort of well-characterized patients. Ferritin, and especially hepcidin, may help identify subjects at risk of critical disease and guide therapeutic optimization to reduce adverse outcomes. Overall, our study confirms the central role of iron metabolism in COVID-19 at the intersection of immune regulation, inflammation, and multiorgan damage.

## Figures and Tables

**Table 1:** Population characteristics at admission according to COVID-19 severity.

Outcome	All (389)	High Severity (172)	Low/Medium Severity (217)	*p* Value
**Age (years)**	71 (61–80)	73 (63–82)	69 (59–78.5)	0.02 *
**Sex (Male percentage)**	258 (66.3%)	112 (65.1%)	146 (67.3%)	0.67
**Inpatient days**	11 (6–20)	17 (10–27)	8 (5–13)	<0.0001 *
**Albumin (g/L)**	34.8 ± 4.6	33.8 ± 4.7	35.9 ± 4.3	<0.001 *
**Bilirubin (mg/dL)**	0.5 (0.3–0.6)	0.5 (0.3–0.6)	0.4 (0.3–0.6)	0.98
**Creatinine (mg/dL)**	0.9 (0.71–1.04)	0.9 (0.7–1)	0.8 (0.7–1)	0.36
**Crp (mg/L)**	72.5 (42.3–128.8)	89 (42–149)	67 (42.5–116)	0.008 *
**D-Dimer (g/L)**	1059 (705–1761)	1069 (736.8–1779.3)	1047 (686–1631)	0.5
**Fibrinogen (g/L)**	5.6 (4.8–6.8)	5.86 (4.93–7.16)	5.52 (4.56–6.55)	0.05 *
**Hemoglobin (Hb) (g/dL)**	13.4 (12.20–14.30)	13.4 (12.2–14.5)	13.3 (12.4–14.2)	0.65
**MCHC (g/L)**	332 (323–339)	329 (321–340)	332 (324–339)	0.3
**MCV (fL)**	90 (86.8–93.65)	90.6 (87–94.1)	89.2 (86.8–93.3)	0.13
**RBC (10^12^/L)**	4.5 ± 0.6	4.5 ± 0.7	4.5 ± 0.6	0.79
**RDW (%)**	13.2 (12.6–14.3)	13.4 (12.8–14.6)	13.1 (12.5–14.2)	0.014 *
**Neutrophils (10^9^/L)**	5.7 (3.8–8.8)	6.3 (4.1–9.6)	5.4 (3.7–7.8)	<0.001 *
**Lymphocytes (10^9^/L)**	0.7 (0.5–1.1)	0.6 (0.5–1)	0.8 (0.6–1.1)	<0.001 *
**N/L ratio**	7.3 (4.2–1)	9.2 (5–17.3)	6.5 (3.7–10.1)	<0.001 *
**WBCs (10^9^/L)**	7.1 (5.0–10.2)	7.7 (5.2–11.2)	6.8 (4.9–9.6)	0.03 *
**Platelets (10^9^/L)**	208 (159–268)	199 (157–258)	215 (163–276)	0.17
**PaO_2_/FiO_2_ ratio**	238 (136–290)	139 (95–236)	271 (233–305)	<0.0001 *
**CCI**	3.00 (2.00–4.00)	3.00 (1.00–4.00)	3.00 (2.00–4.00)	0.051
**Treatment**				
**Antivirals**	34.5%	28%	35.4%	0.098
**Heparins**	91.7%	86.6%	84.3%	1
**Steroids**	98.9%	94.2%	92.2%	0.2
**Tocilizumab**	0.01%	0.01%	0.01%	-

Data are presented as n (%) for categorical variables and as medians (25th –75th percentiles) for continuous variables. Chi-squared test and Mann-Whitney test between severity groups were used for calculating the *p*-value for categorical and continuous variables respectively. “*” statistically significant. CCI = Charlson Comorbidity Index, CRP = C Reactive Protein; MCV = Mean Corpuscular Volume; MCHC = Mean Corpuscular Hemoglobin Concentration; N/L ratio = Neutrophils over Lymphocytes ratio; RBC = Red Blood Cells; PaO_2_/FiO_2_ ratio or P/F ratio = ratio of arterial Oxygen Partial Pressure to Fractional inspired Oxygen; WBCs = White Blood Cell Count; RDW = Red Blood Cell Distribution Width.

**Table 2:** Iron-related parameters at admission according to COVID-19 severity.

Outcome	All (389)	High Severity (172)	Low/Medium Severity (217)	*p* Value
**TSAT (%)**	16 (10.8–27)	16 (10–27)	17 (11–26)	0.75
**Transferrin (g/L)**	1.6 (1.4–1.9)	1.6 (1.3–1.9)	1.6 (1.4–1.9)	0.31
**Ferritin (µg/L)**	861.5 (439.8–1346.8)	965.5 (573.8–1623)	787 (386.5–1197.5)	0.001 *
**Iron (µg/dL)**	34.6 (24–56.9)	33.2 (21.9–51.4)	36.6 (24.3–59.6)	0.18
**Erfe (ng/mL)**	0.4 (0.1–1.1)	0.5 (0.1–1.2)	0.4 (0.1–1)	0.097
**Hepcidin (ng/mL)**	201 (130.3–265.2)	219.2 (137–297.1)	189 (124.4–246)	0.007 *
**sTfR (ng/mL)**	1738.6 (1415.9–2220.5)	1872.1 (1434.5–2428.1)	1667 (1392.8–2050.4)	0.026 *

Data are presented as medians (25th–75th percentiles) for continuous variables. Mann-Whitney test between severity groups was used for calculating the *p*-value “*” statistically significant. TSAT = Transferrin saturation; sTfR = soluble transferrin receptor; ERFE = erythroferrone.

**Table 3:** Cytokine profile at admission according to COVID-19 severity.

Outcome	All (389)	High Severity (172)	Low/Medium Severity (217)	*p* Value
**IFN-γ (pg/mL)**	3.6 (1.1–11.9)	3.1 (0.9–9.5)	3.8 (1.3–15.9)	0.15
**IL-10 (pg/mL)**	13.2 (7.6–24.7)	16 (9.7–30.2)	11.2 (6–20.7)	<0.001 *
**IL-1-β (pg/mL)**	0.3 (0.2–0.5)	0.2 (0.1–0.6)	0.3 (0.2–0.5)	0.1
**IL1-RA (pg/mL)**	1190 (746–2361)	1310 (801–2373)	1112 (665–2243)	0.06
**IL-22 (pg/mL)**	16.9 (10.2–26.8)	16.4 (9.4–28.4)	17.7 (10.4–26.2)	0.5
**IL-6 (pg/mL)**	22.6 (9.2–50.7)	29.1 (12.9–65.8)	16.8 (6.1–43.7)	<0.001 *
**IL-8 (pg/mL)**	7.9 (4.9–11.8)	8.9 (6.1–13.4)	6.8 (4.6–10.6)	<0.001 *
**TNF-α (pg/mL)**	13.2 (10.5–17.4)	13 (10.5–17.4)	13.7 (10.5–17.4)	0.86

Data are presented as medians (25th–75th percentiles) for continuous variables. Mann-Whitney test between severity groups was used for calculating the *p*-value. “*” statistically significant.

## Data Availability

The data presented in this study are available on request from the corresponding author.

## Supplementary Materials

The following supporting information can be downloaded at: https://www.mdpi.com/article/10.3390/nu18213570/s1, Supplementary Material Figure S1. Flowchart of patient stratification based on clinical outcomes and respiratory support. Figure S2. Forest plot showing the odds ratios and 95% confidence intervals for predictors of disease severity. Figure S3. Correlation heatmap of iron metabolism, inflammatory cytokines, and clinical parameters. Figure S4. Incremental predictive value of clinical and biological blocks on COVID-19 severity. Figure S5. Hepcidin-IL6-Ferritin axis in severe COVID-19 disease. Table S1. Population characteristics at admission according to COVID-19 severity (restricted to patients who completed all three follow-up points). Table S2. Population characteristics at T1 according to COVID-19 severity (restricted to patients who completed all three follow-up points). Table S3. Population characteristics at T2 according to COVID-19 severity (restricted to patients who completed all three follow-up points). Table S4. Iron-related parameters at T0 according to COVID-19 severity (restricted to patients who completed all three follow-up points). Table S5. Iron-related parameters at T1 according to COVID-19 severity (restricted to patients who completed all three follow-up points). Table S6. Iron-related parameters at T2 according to COVID-19 severity (restricted to patients who completed all three follow-up points). Table S7. Cytokine profile at T0 according to COVID-19 severity (restricted to patients who completed all three follow-up points). Table S8. Cytokine profile at T1 according to COVID-19 severity (restricted to patients who completed all three follow-up points). Table S9. Cytokine profile at T2 according to COVID-19 severity (restricted to patients who completed all three follow-up points). Table S10. Logistic regression model for COVID-19 severity. Table S11. Logistic regression model for COVID-19 severity. Table S12. Multivariate Linear Regression Analysis. Table S13. Multivariate Linear Regression Analysis. Table S14. Logistic regression model for COVID-19 severity.

## Abbreviations

The following abbreviations are used in this manuscript:

ALP	Alkaline Phosphatase
AST	Aspartate Aminotransferase
CRP	C Reactive Protein
eGFR	Estimated Glomerular Filtration Rate
ERFE	Erythroferrone
FT	Ferritin
FPN	Ferroportin
Hb	Hemoglobin
HT	Hematocrit
IFN	Interferon
IL	Interleukin
LDH	Lactate Dehydrogenase
MCHC	Mean Corpuscular Hemoglobin Concentration
MCV	Mean Corpuscular Volume
N/L ratio	Neutrophils over Lymphocytes ratio
PaO_2_/FiO_2_ ratio or P/F ratio	ratio of arterial Oxygen Partial Pressure to Fractional inspired Oxygen
RBC	Red Blood Cells
RDW	Red Blood Cell Distribution Width
sTfR	Soluble Transferrin Receptor
TNF	Tumor Necrosis Factor
TSAT	Transferrin Saturation
WBCs	White Blood Cell Count
