# Supplementary material for: Iron Dysregulation During Severe COVID-19: Longitudinal Evidence from Hospitalized Patients
Source: Nutrients. 2026 Oct 29;18(21):3570. doi: 10.3390/nu18213570 (PMC13765291; doi:10.3390/nu18213570)
Supplement: Supplementary file 1 [file nutrients-18-03570-s001.zip › nutrients-4573650-supplementary.pdf]

SUPPLEMENTARY MATERIAL

SUPPLEMENTARY FIGURES:

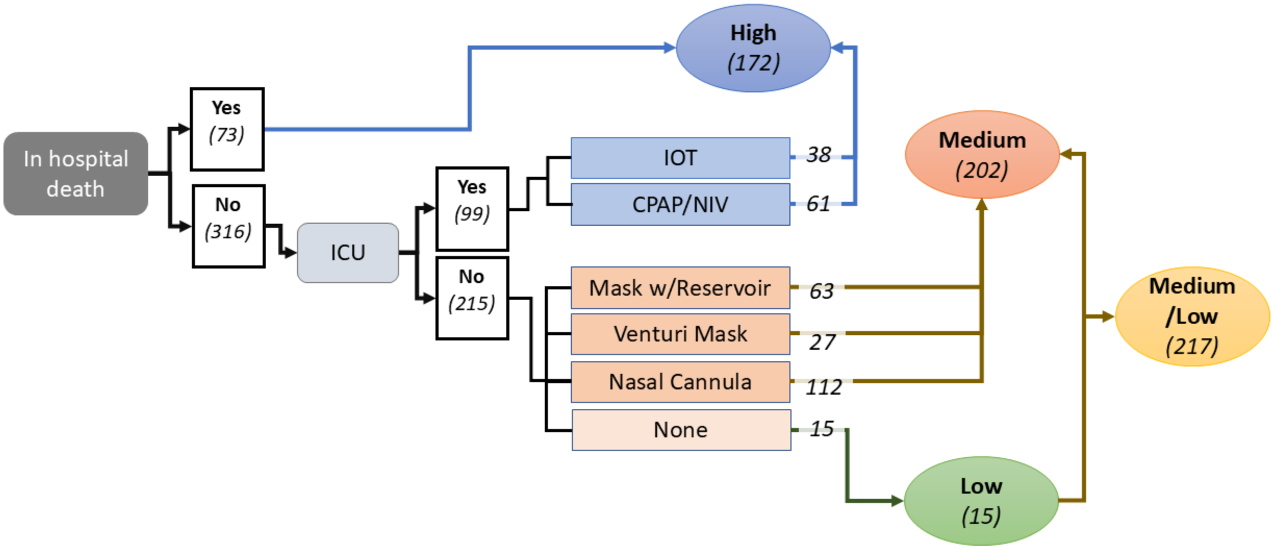

Figure S1. Flowchart of patient stratification based on clinical outcomes and respiratory support. The study population was categorized into three severity levels: High (including in-hospital deaths and patients requiring IOT or CPAP/NIV), Medium (patients treated with reservoir, Venturi, or nasal cannula), and Low (patients requiring no supplemental oxygen). The
"Medium/Low" subgroup (n=217) combines patients with non-invasive oxygen therapy or no support. IOT= Orotracheal Intubation; CPAP/NIV= Continuous Positive Airway Pressure/Non-Invasive Ventilation.

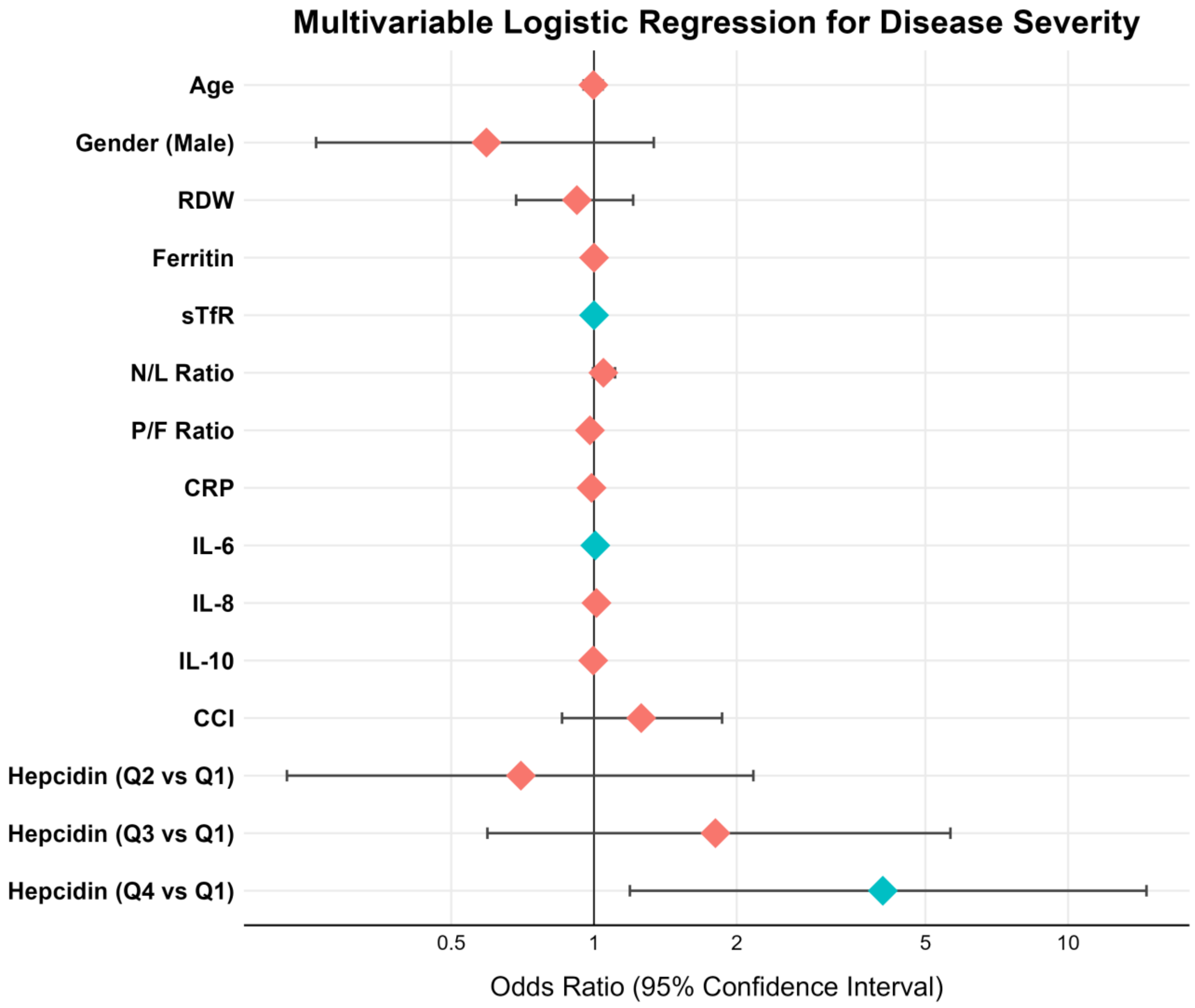

Figure S2 -Forest plot showing the odds ratios and 95% confidence intervals for predictors of disease severity. Hepcidin levels are represented in quartiles, with Q1 as the reference group. I quartile (Q1): hepcidin<12.5 ng/mL; II quartile(Q2): 129.5 ng/mL<hepcidin< 200.9 ng/mL, III quartile(Q3): 200.9 ng/mL <hepcidin <265.3 ng/mL; IV quartile(Q4): hepcidin >265.3 ng/mL. Orange dots represent variable with p>0.05; blue dots represent variable with p<0.05.RDW= Red Blood Cell Distribution Width; CRP= C-reactive protein, CCI=Charlson Comorbidity Index.

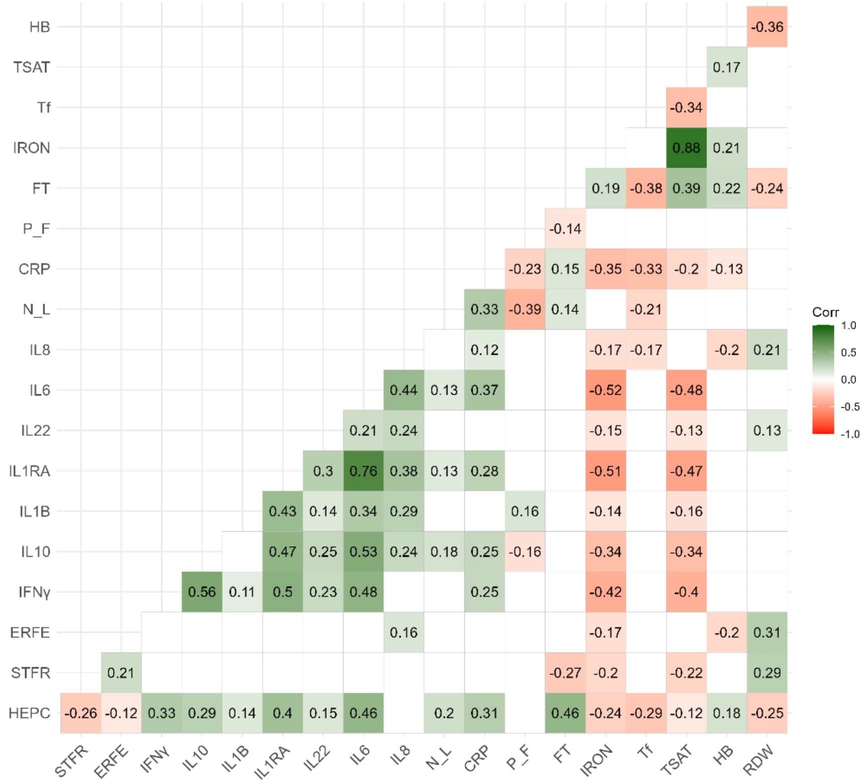

Figure S3 -Correlation heatmap of iron metabolism, inflammatory cytokines, and clinical parameters. The triangular matrix displays Spearman's correlation coefficients (r) between all analyzed variables. Green cells indicate a positive correlation, while red cells indicate a negative correlation. The intensity of color and numerical values represents the strength of the association. White cells denote non-significant correlations ($p > 0.05$). HEPC= Hepcidin; N_L= Neutrophils/ Lymphocytes ratio; CRP= C-Reactive Protein; P_F= PaO_2/FiO_2 ratio; FT= Ferritin; Tf= Transferrin; TSAT= Transferrin Saturation; HB= Hemoglobin; RDW= Red Blood Cell Distribution Width.

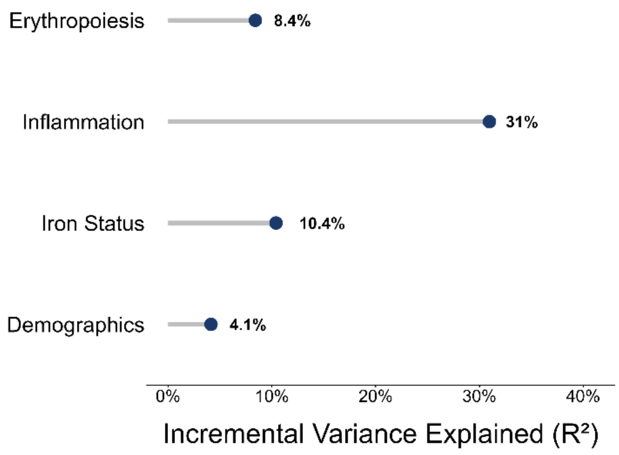

Figure S4-Incremental predictive value of clinical and biological blocks on COVID-19 severity. The lollipop chart illustrates the change in variance explained (ΔR^2) by sequentially adding blocks of variables to the regression model. Block I-demographics: Age, gender; Block II- Iron markers (serum iron, sTfR); Block II- Inflammatory markers: CRP, Ft, and cytokines; Block IV-erythropoiesis-related factors: ERFE, Hb, RDW, P/F. Block III (inflammation) accounts for the largest incremental contribution to the model's predictive power.

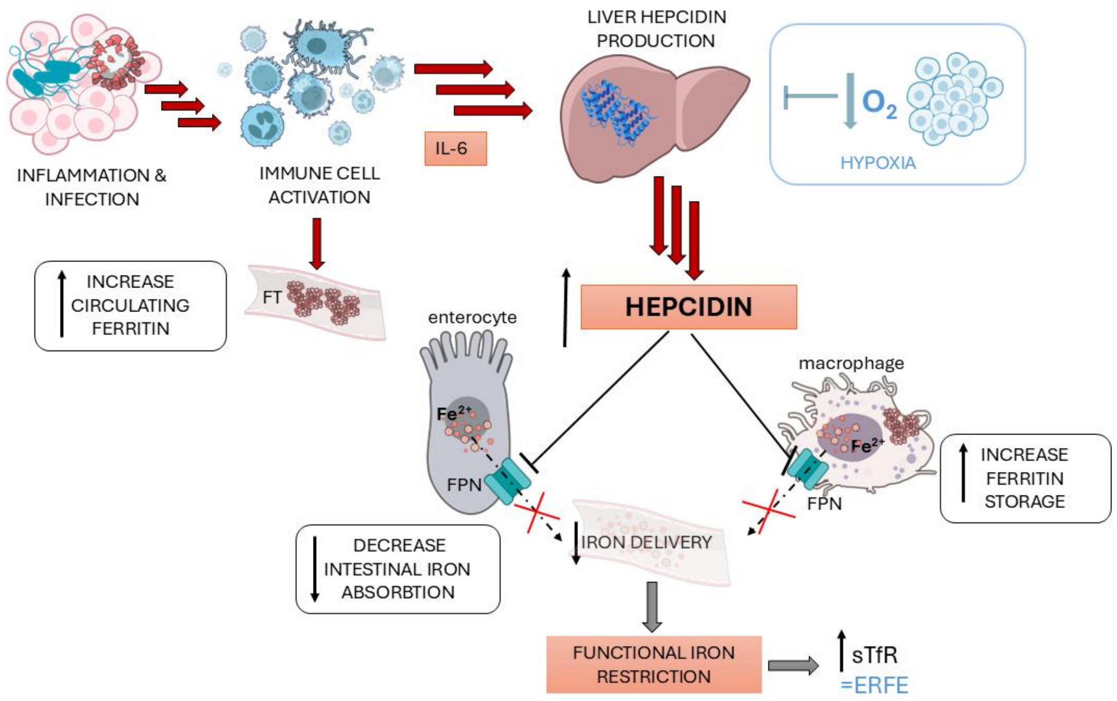

Figure S5- Hepcidin-IL6-Ferritin axis in severe COVID-19 disease. The schematic diagram illustrates the behavior of IL-6, Hepcidin, and Ferritin based on our analysis of the study population. We hypothesize that inflammation is the primary driver of the system, overriding the concomitant effects of hypoxia, iron restriction, and ineffective erythropoiesis in these patients. Elevated hepcidin levels, induced by IL-6 stimulus, lead to the blockage of FPN and consequently decreased iron absorption, increased stored and circulating ferritin (by inflammation itself), decreased iron delivery, and cause increased sTfR. Items in light blue indicate an expected inhibitory role on hepcidin. IL-6: interleukin 6; FT: ferritin; sTfR: soluble transferrin receptor; ERFE: erythroferrone; FPN: ferroportin.

SUPPLEMENTARY TABLES

Table S1. Population characteristics at admission according to COVID-19 severity (restricted to patients who completed all three follow-up points). All variables are presented as medians (25th - 75th). The p-value was calculated using the Mann-Whitney test between severity groups. "*" indicates statistically significant p values. CRP= C Reactive Protein. MCHC= Mean Corpuscular Hemoglobin Concentration. MCV= Mean Corpuscular Volume. N/L ratio= Neutrophils over Lymphocytes ratio. PaO2/FiO2 ratio or P/F ratio= ratio of arterial Oxygen Partial Pressure to Fractional inspired Oxygen. RBC= Red Blood Cells. RDW= Red Blood Cell Distribution Width. WBCs= White Blood Cell Count.

OUTCOME	ALL (91)	LOW/MEDIUM SEVERITY (25)	HIGH SEVERITY (66)	P VALUE
**ALBUMIN (G/L)**	33.25 (31.10 - 37.30)	35.40 (31.10 - 38.20)	32.90 (30.70 - 36.90)	0.14
**CREATININE (MG/DL)**	0.88 (0.74 - 1.06)	0.98 (0.78 - 1.11)	0.87 (0.72 - 1.01)	0.2
**CRP (MG/L)**	102.00 (42.00 - 181.00)	104.50 (41.50 - 183.50)	97.00 (42.00 - 181.00)	0.9
**D-DIMER (G/L)**	1,258.00 (762.00 - 1,806.00)	1,683.50 (1,310.00 - 2,204.00)	1,001.00 (685.00 - 1,765.00)	0.046*
**FIBRINOGEN (G/L)**	5.77 (4.82 - 7.24)	5.33 (4.47 - 7.05)	5.88 (4.93 - 7.34)	0.5
**HEMOGLOBIN (G/DL)**	13.30 (12.20 - 14.20)	12.25 (11.00 - 13.25)	13.60 (12.40 - 14.40)	0.003*
**HEMATOCRIT (%)**	41.00 (37.00 - 43.00)	39.00 (33.00 - 41.00)	41.00 (38.00 - 43.50)	0.030*
**LYMPHOCYTES (10^9/L)**	0.69 (0.47 - 1.07)	0.90 (0.59 - 1.06)	0.63 (0.45 - 1.07)	0.2
**MCHC (G/L)**	330.50 (323.00 - 339.00)	324.50 (319.00 - 336.00)	333.00 (324.00 - 340.00)	0.056
**MCV (FL)**	90.35 (87.30 - 94.00)	91.45 (89.05 - 95.55)	89.75 (87.00 - 93.50)	0.11
**N/L**	8.12 (4.75 - 15.39)	5.21 (2.68 - 8.43)	9.31 (5.24 - 17.16)	0.004*
**NEUTROPHILS (10^9/L)**	5.93 (4.08 - 9.05)	4.62 (2.43 - 6.81)	6.23 (4.40 - 9.37)	0.032*
**PAO2/FIO2 RATIO**	168.50 (108.00 - 281.00)	288.00 (250.00 - 345.50)	130.50 (95.00 - 220.00)	<0.001*
**PLATELETS (10^9/L)**	195.50 (154.00 - 255.00)	180.00 (154.50 - 219.00)	210.50 (153.00 - 257.00)	0.14
**RBC (10^12/L)**	4.42 (4.04 - 4.78)	4.17 (3.70 - 4.55)	4.48 (4.15 - 4.89)	0.007*
**RDW (%)**	13.50 (12.80 - 14.40)	14.30 (13.60 - 14.85)	13.15 (12.70 - 14.00)	<0.001*
**WBC (10^9/L)**	7.28 (5.19 - 10.72)	7.22 (4.04 - 11.40)	7.38 (5.50 - 10.72)	0.3

Table S2-Population characteristics at T1 according to COVID-19 severity (restricted to patients who completed all three follow-up points). All variables are presented as medians (25th - 75th). The p-value was calculated using the Mann-Whitney test between severity groups. "*" indicates statistically significant p values. CRP= C Reactive Protein. MCHC= Mean Corpuscular Haemoglobin Concentration. MCV= Mean Corpuscular Volume. N/L ratio= Neutrophils over Lymphocytes ratio. PaO_2/FiO_2 ratio or P/F ratio= ratio of arterial Oxygen Partial Pressure to Fractional inspired Oxygen. RBC= Red Blood Cells. RDW= Red Blood Cell Distribution Width. WBCs= White Blood Cell Count.

OUTCOME	ALL (91)	LOW/MEDIUM SEVERITY (25)	HIGH SEVERITY (66)	P VALUE
ALBUMIN (G/L)	31.20 (28.30 - 32.90)	21.30 (21.30 - 21.30)	31.25 (28.50 - 32.95)	0.10
CREATININE (MG/DL)	0.76 (0.64 - 0.96)	0.88 (0.76 - 1.18)	0.73 (0.61 - 0.87)	0.009*
CRP (MG/L)	44.50 (18.50 - 90.00)	31.00 (18.00 - 74.00)	47.00 (20.00 - 92.00)	0.4
D-DIMER (G/L)	795.00 (376.00 - 1,148.00)	1,230.00 (1,230.00 - 1,230.00)	739.50 (376.00 - 1,129.00)	0.3
FIBRINOGEN (G/L)	5.16 (4.34 - 6.27)			
HEMOGLOBIN (G/DL)	12.35 (11.10 - 13.50)	11.70 (10.50 - 13.00)	12.60 (11.20 - 13.50)	0.2
HEMATOCRIT (%)	38.00 (35.00 - 41.00)	36.00 (33.00 - 40.00)	39.00 (35.00 - 42.00)	0.2
LYMPHOCYTES (10^9/L)	0.95 (0.54 - 1.39)	0.95 (0.89 - 1.41)	0.95 (0.49 - 1.37)	0.3
MCHC (G/L)	328.00 (320.00 - 335.00)	326.00 (320.00 - 334.00)	328.00 (320.00 - 335.00)	0.6
MCV (FL)	91.40 (88.60 - 94.70)	91.40 (88.80 - 94.70)	91.40 (88.60 - 94.30)	>0.9
N/L	9.20 (5.55 - 16.51)	6.08 (4.21 - 9.17)	11.54 (6.46 - 19.33)	0.005*
NEUTROPHILS (10^9/L)	9.19 (6.75 - 11.23)	7.48 (3.75 - 10.94)	9.61 (7.16 - 11.25)	0.061
PAO2/FIO2 RATIO	170.00 (122.00 - 246.00)	146.50 (85.50 - 242.00)	170.00 (125.00 - 246.00)	0.6
PLATELETS (10^9/L)	245.50 (200.00 - 331.00)	231.00 (183.00 - 280.00)	263.00 (206.00 - 354.00)	0.10
RBC (10^12/L)	4.19 (3.68 - 4.64)	3.92 (3.61 - 4.53)	4.31 (3.74 - 4.66)	0.2
RDW (%)	13.25 (12.50 - 14.20)	14.10 (13.10 - 14.80)	13.10 (12.50 - 13.90)	0.024*
WBC (10^9/L)	11.45 (8.49 - 13.44)	9.81 (5.39 - 12.89)	11.51 (8.95 - 13.44)	0.3

Table S3-Population characteristics at T2 according to COVID-19 severity (restricted to patients who completed all three follow-up points). All variables are presented as medians (25th - 75th). The p-value was calculated using the Mann-Whitney test between severity groups. "*" indicates statistically significant p values. CRP= C Reactive Protein. MCHC= Mean Corpuscular Haemoglobin Concentration. MCV= Mean Corpuscular Volume. N/L ratio= Neutrophils over Lymphocytes ratio. PaO2/FiO2 ratio or P/F ratio= ratio of arterial Oxygen Partial Pressure to Fractional inspired Oxygen. RBC= Red Blood Cells. RDW= Red Blood Cell Distribution Width. WBCs= White Blood Cell Count.

OUTCOME	ALL (91)	LOW/MEDIUM SEVERITY (25)	HIGH SEVERITY (66)	P VALUE
ALBUMIN (G/L)	30.85 (27.90 - 33.20)	27.40 (27.40 - 27.40)	30.90 (28.30 - 33.20)	0.3
CREATININE (MG/DL)	0.84 (0.64 - 1.02)	0.91 (0.79 - 1.04)	0.78 (0.60 - 1.01)	0.12
CRP (MG/L)	44.00 (14.00 - 114.00)	24.00 (12.00 - 74.50)	50.00 (15.00 - 121.00)	0.2
D-DIMER (G/L)	962.00 (583.00 - 1,042.00)	583.00 (583.00 - 583.00)	972.50 (579.50 - 1,217.50)	0.4
FIBRINOGEN (G/L)	5.85 (4.93 - 7.44)	4.17 (4.17 - 4.17)	6.03 (5.11 - 7.44)	0.2
HEMOGLOBIN (G/DL)	11.40 (10.20 - 12.50)	11.10 (10.50 - 12.30)	11.40 (10.00 - 12.60)	>0.9
HEMATOCRIT (%)	35.00 (32.00 - 38.00)	35.00 (32.00 - 38.00)	35.00 (32.00 - 40.00)	0.9
LYMPHOCYTES (10^9/L)	1.33 (0.78 - 1.90)	1.47 (1.09 - 1.89)	1.18 (0.69 - 2.06)	0.3
MCHC (G/L)	325.00 (317.00 - 332.00)	324.00 (316.00 - 330.00)	326.00 (317.00 - 332.00)	0.4
MCV (Fl)	92.40 (89.80 - 95.00)	92.60 (88.50 - 96.50)	92.00 (90.60 - 94.90)	0.8
N/L	5.84 (3.66 - 10.91)	4.09 (2.57 - 6.59)	7.17 (4.08 - 16.84)	0.004*
NEUTROPHILS (10^9/L)	8.25 (5.21 - 11.21)	6.08 (4.32 - 7.40)	9.10 (7.10 - 13.01)	<0.001*
PaO2/FiO2 ratio	218.00 (131.00 - 279.00)	238.00 (214.00 - 262.00)	218.00 (131.00 - 279.00)	0.8
PLATELETS (10^9/L)	204.00 (149.00 - 282.00)	229.00 (173.00 - 257.00)	200.00 (144.00 - 287.00)	0.6
RBC (10^9/L)	3.86 (3.38 - 4.24)	3.77 (3.50 - 4.17)	3.89 (3.38 - 4.26)	>0.9
RDW (%)	13.65 (13.00 - 14.90)	14.80 (13.90 - 15.30)	13.30 (12.80 - 14.50)	0.006*
WBC (10^9/L)	11.02 (8.11 - 14.81)	9.12 (6.66 - 10.50)	11.66 (9.63 - 15.89)	0.001*

Table S4- Iron-related parameters at T0 according to COVID-19 severity (restricted to patients who completed all three follow-up points). All variables are presented as medians (25th - 75th). The p-value was calculated using the Mann-Whitney test between severity groups. "*" indicates statistically significant p values. TSAT= Transferrin saturation. ERFE= erythroferrone. HEPC= hepcidin. sTfR=soluble transferrin receptor.

OUTCOME	ALL (91)	LOW/MEDIUM SEVERITY (25)	HIGH SEVERITY (66)	P VALUE
TSAT (%)	16.00 (10.00 - 27.00)	15.00 (9.50 - 23.50)	16.00 (10.00 - 27.00)	0.6
TRANSFERRIN (g/L)	1.59 (1.32 - 1.93)	1.91 (1.28 - 2.08)	1.58 (1.35 - 1.82)	0.3
FERRITIN (µg/L)	1,006.00 (536.00 - 1,583.00)	839.00 (298.00 - 1,213.00)	1,068.00 (669.50 - 1,648.00)	0.031*
IRON (µg/dL)	32.59 (23.12 - 52.83)	29.01 (22.84 - 48.08)	35.60 (23.40 - 64.56)	0.7
ERFE (ng/mL)	0.42 (0.14 - 1.20)	0.39 (0.14 - 1.32)	0.43 (0.14 - 1.20)	0.8
HEPC (ng/mL)	230.39 (135.73 - 321.22)	182.29 (111.45 - 252.76)	251.57 (141.06 - 336.80)	0.024*
STFR (ng/mL)	1,668.69 (1,343.73 - 2,165.62)	1,556.75 (1,278.53 - 2,017.13)	1,697.54 (1,388.08 - 2,224.28)	0.14

Table S5- Iron-related parameters at T1 according to COVID-19 severity (restricted to patients who completed all three follow-up points). All variables are presented as medians (25th - 75th). The p-value was calculated using the Mann-Whitney test between severity groups. "*" indicates statistically significant p values. TSAT= Transferrin saturation. ERFE= erythroferrone. HEPC= hepcidin. sTfR=soluble transferrin receptor.

OUTCOME	ALL (91)	LOW/MEDIUM SEVERITY (25)	HIGH SEVERITY (66)	P VALUE
TSAT (%)	36.00 (25.00 - 50.00)	31.00 (19.50 - 44.50)	37.00 (29.00 - 53.00)	0.2
TRANSFERRIN (g/L)	1.37 (1.17 - 1.86)	1.49 (1.25 - 1.96)	1.37 (1.12 - 1.85)	0.5
FERRITIN (µg/L)	1,167.50 (804.00 - 2,051.00)	821.00 (465.00 - 1,881.00)	1,286.00 (930.00 - 2,229.00)	0.042*
IRON (µg/dL)	69.11 (47.58 - 107.34)	55.93 (35.19 - 113.59)	70.59 (53.59 - 100.41)	0.3
ERFE (ng/mL)	0.14 (0.14 - 0.54)	0.53 (0.14 - 0.81)	0.14 (0.14 - 0.28)	0.021*
HEPC (ng/mL)	188.43 (131.18 - 261.38)	134.89 (100.54 - 206.33)	197.16 (146.12 - 275.28)	0.007*
STFR (ng/mL)	1,287.79 (883.77 - 1,663.62)	1,460.23 (1,008.75 - 2,247.28)	1,195.95 (883.77 - 1,546.22)	0.073

Table S6-Iron-related parameters at T2 according to COVID-19 severity (restricted to patients who completed all three follow-up points). All variables are presented as medians (25th - 75th). The p-value was calculated using the Mann-Whitney test between severity groups. The p-value was calculated using the Mann-Whitney test between severity groups. "*" indicates statistically significant p values. TSAT= Transferrin saturation. ERFE= erythroferrone. HEPC= hepcidin. sTfR=soluble transferrin receptor.

OUTCOME	ALL (91)	LOW/MEDIUM SEVERITY (25)	HIGH SEVERITY (66)	P VALUE
**TSAT (%)**	32.00 (19.00 - 43.00)	25.00 (17.50 - 34.04)	36.00 (23.00 - 45.00)	0.027*
**TRANSFERRIN (g/L)**	1.39 (1.15 - 1.78)	1.61 (1.30 - 1.74)	1.33 (1.02 - 1.79)	0.14
**FERRITIN (µg/L)**	1,008.50 (548.00 - 1,638.00)	537.50 (348.50 - 1,126.50)	1,129.50 (701.50 - 1,864.00)	0.005*
**IRON (µg/dL)**	61.43 (38.42 - 84.52)	56.46 (34.74 - 78.44)	63.41 (41.16 - 90.54)	0.2
**ERFE (ng/mL)**	0.38 (0.14 - 1.09)	0.57 (0.14 - 0.97)	0.34 (0.14 - 1.26)	0.5
**HEPC (ng/mL)**	151.01 (80.75 - 217.76)	87.09 (51.73 - 175.28)	157.36 (107.11 - 241.97)	0.011*
**STFR (ng/mL)**	1,111.49 (815.13 - 1,420.82)	1,210.47 (1,107.06 - 1,464.77)	973.07 (709.11 - 1,336.39)	0.030*

Table S7- Cytokine profile at T0 according to COVID-19 severity (restricted to patients who completed all three follow-up points). All variables are presented as medians (25th - 75th). The p-value was calculated using the Mann-Whitney test. "*" indicates statistically significant p values.

OUTCOME	ALL (91)	LOW/MEDIUM SEVERITY (25)	HIGH SEVERITY (66)	P VALUE
**IFN-γ (pg/mL)**	3.64 (0.90 - 9.52)	3.71 (0.96 - 17.60)	2.70 (0.83 - 8.71)	0.4
**IL-10 (pg/mL)**	16.00 (10.00 - 30.60)	17.20 (9.95 - 29.85)	15.50 (10.00 - 31.50)	0.8
**IL-1-β (pg/mL)**	0.28 (0.13 - 0.52)	0.33 (0.12 - 0.50)	0.27 (0.13 - 0.56)	0.9
**IL1-R-A (pg/mL)**	1,310.00 (751.00 - 2,169.00)	1,707.50 (778.50 - 2,808.50)	1,137.00 (751.00 - 1,903.00)	0.3
**IL-22 (pg/mL)**	16.00 (8.75 - 27.00)	23.20 (14.15 - 47.15)	14.00 (8.05 - 22.90)	0.010*
**IL-6 (pg/mL)**	27.70 (11.20 - 63.20)	33.10 (10.75 - 63.80)	27.70 (11.20 - 61.90)	0.9
**IL-8 (pg/mL)**	8.25 (6.44 - 11.30)	8.95 (5.91 - 16.00)	8.12 (6.48 - 11.10)	0.6
**TNF-α (pg/mL)**	1.59 (1.32 - 1.93)	15.55 (13.05 - 20.70)	12.00 (10.50 - 16.20)	0.004*

Table S8- Cytokine profile at T1 according to COVID-19 severity (restricted to patients who completed all three follow-up points). All variables are presented as medians (25th - 75th). The p-value was calculated using the Mann-Whitney test. "*" indicates statistically significant p values.

OUTCOME	ALL (91)	LOW/MEDIUM SEVERITY (25)	HIGH SEVERITY (66)	P VALUE
**IFN-γ (pg/mL)**	0.93 (0.35 - 1.90)	1.19 (0.77 - 2.16)	0.88 (0.28 - 1.90)	0.13
**IL-10 (pg/mL)**	7.78 (4.88 - 16.40)	8.67 (4.83 - 14.85)	7.61 (4.88 - 18.70)	0.6
**IL-1-β (pg/mL)**	0.37 (0.20 - 0.66)	0.30 (0.10 - 1.05)	0.41 (0.21 - 0.66)	0.7
**IL1-R-A (pg/mL)**	1,283.50 (875.00 - 2,573.00)	1,172.50 (667.00 - 2,469.50)	1,302.50 (966.00 - 2,573.00)	0.4
**IL-22 (pg/mL)**	16.20 (7.01 - 43.70)	45.35 (10.50 - 56.05)	14.10 (6.99 - 29.60)	0.032*
**IL-6 (pg/mL)**	22.70 (8.57 - 65.60)	23.40 (8.26 - 38.60)	22.25 (9.39 - 70.80)	0.5
**IL-8 (pg/mL)**	8.47 (6.48 - 11.20)	8.40 (6.13 - 10.08)	8.89 (6.48 - 12.10)	0.7
**TNF-α (pg/mL)**	1.37 (1.17 - 1.86)	15.40 (12.75 - 18.55)	15.20 (12.20 - 20.30)	0.8

Table S9-Cytokine profile at T2 according to COVID-19 severity (restricted to patients who completed all three follow-up points). All variables are presented as medians (25th - 75th). The p-value was calculated using the Mann-Whitney test. "*" indicates statistically significant p values.

OUTCOME	ALL (91)	LOW/MEDIUM SEVERITY (25)	HIGH SEVERITY (66)	P VALUE
**IFN-γ (pg/mL)**	1.15 (0.53 - 2.52)	1.79 (1.27 - 4.56)	0.78 (0.43 - 2.02)	0.002*
**IL-10 (pg/mL)**	6.04 (4.06 - 10.00)	6.59 (5.11 - 9.47)	5.92 (3.96 - 10.50)	0.5
**IL-1-β (pg/mL)**	0.52 (0.26 - 1.01)	0.52 (0.33 - 0.93)	0.52 (0.26 - 1.01)	0.7
**IL1-R-A (pg/mL)**	1,740.00 (975.00 - 3,316.00)	1,670.50 (686.00 - 3,779.00)	1,740.00 (1,156.00 - 3,035.00)	0.7
**IL-22 (pg/mL)**	17.50 (7.58 - 36.10)	27.55 (11.20 - 44.80)	15.40 (6.64 - 28.20)	0.080
**IL-6 (pg/mL)**	19.30 (10.10 - 46.20)	19.00 (13.00 - 36.90)	19.30 (9.79 - 60.30)	0.5
**IL-8 (pg/mL)**	8.40 (6.22 - 13.50)	6.62 (6.22 - 10.40)	8.86 (6.25 - 14.00)	0.15
**TNF-α (pg/mL)**	1.39 (1.15 - 1.78)	18.85 (15.70 - 23.80)	15.40 (11.20 - 22.80)	0.2

Table S10-Logistic regression model for COVID-19 severity. Dependent variable: severity. Independent variables: age, gender, ferritin, N/L ratio, IL-10, IL-6, IL-8, RDW, CRP, P/F, hepcidin, therapy and CCI. Beta=unstandardized regression coefficient indicates the magnitude of effect. Exp(B)= Odds Ratio, if Exp(B) > 1, the odds of the outcome increase as the predictor increases. P value indicates whether the predictor is statistically significant. "*" indicates statistically significant p values. P/F ratio= ratio of arterial Oxygen Partial Pressure to Fractional inspired Oxygen; RDW= Red Blood Cell Distribution Width; N/L= Neutrophils over Lymphocytes ratio; CRP= C-reactive protein; FT= Ferritin, CCI= Charlson Comorbidity Index.

	BETA (B)	OR (EXP(B))	P VALUE
**AGE**	0.00	1.00	0.9
**GENDER (M)**	-0.50	0.60	0.2
**RDW**	-0.04	0.96	0.8
**FT**	0.00	1.00	0.2
**HEPC**	0.01	1.01	0.004*
**IL-10**	0.00	1.00	0.7
**IL-6**	0.01	1.01	0.041*
**IL-8**	0.01	1.01	0.059
**STFR**	0.00	1.00	0.026*
**N/L**	0.05	1.05	0.10
**CRP**	-0.01	0.99	<0.001*
**P/F**	-0.02	0.98	<0.001*
**CCI**	0.24	1.28	0.2

Table S11-Logistic regression model for COVID-19 severity. Dependent variable: severity. Independent variables: age, gender, ferritin, N/L ratio, IL-10, IL-6, IL-8, RDW, CRP, P/F, hepcidin percentiles. I quartile or reference group: hepcidin<129,5 ng/mL; II quartile: 129,5 ng/mL<hepcidin< 200,9 ng/mL, III quartile: 200,9 ng/mL <hepcidin <265,3 ng/mL; IV quartile: hepcidin >265,3 ng/mL. Beta=unstandardized regression coefficient, indicates the magnitude of effect. Exp(B)= Odds Ratio, if Exp(B) > 1, the odds of the outcome increase as the predictor increases. P value indicates whether the predictor is statistically significant. "*" indicates statistically significant p values.P/F ratio= ratio of arterial Oxygen Partial Pressure to Fractional inspired Oxygen; RDW= Red Blood Cell Distribution Width; N/L= Neutrophils over Lymphocytes ratio; CRP= C-reactive protein; FT= Ferritin, CCI= Charlson Comorbidity Index.

	BETA (B)	OR (EXP(B))	P VALUE
**AGE**	-0.003	0.997	0.9
**GENDER(M)**	-0.522	0.593	0.21
**RDW**	-0.083	0.92	0.568
**FT**	0	1	0.175
**HEPCIDIN-II quartile**	-0.355	0.701	0.537
**HEPCIDIN-III quartile**	0.59	1.804	0.301
**HEPCIDIN-IV quartile**	1.403	4.066	0.028*
**IL-10**	-0.004	0.996	0.546
**IL-6**	0.006	1.006	0.034*
**IL-8**	0.012	1.012	0.102
**STFR**	0	1	0.04*
**N/L**	0.046	1.047	0.093
**CRP**	-0.012	0.988	<0.001*
**P/F**	-0.02	0.981	<0.001*
**CCI**	0.229	1.258	0.24

Table S12. Multivariate Linear Regression Analysis. Dependent variable: serum iron. Independent variables: age, gender, Hb, RDW, FT, N/L ratio, ERFE, sTfR IL10, IL6, IL8, IL1-RA, hepcidin, transferrin, P/F ratio and CCI=Charlson Comorbidity Index. Std. Beta = standardized regression coefficient, compares the relative importance of predictors. CI=Confidence Interval. P value indicates whether the predictor is statistically significant. "*" indicates statistically significant p values. RDW= Red Blood Cell Distribution Width. FT= Ferritin, N/L ratio= Neutrophils over Lymphocytes ratio.
ERFE=erythroferrone. sTfR= Soluble Transferrin Receptor. P/F ratio= ratio of arterial Oxygen Partial Pressure to Fractional inspired Oxygen.

	**STD. BETA**	**95% CI**	**P VALUE**
**AGE**	-0.17	-0.61, 0.26	0.4
**GENDER [M]**	1.3	-5.8, 8.5	0.7
**RDW**	0.15	-2.0, 2.3	0.9
**HB**	1.7	-0.43, 3.8	0.12
**FT**	0.01	0.00, 0.01	0.004*
**N/L**	0.41	-0.04, 0.85	0.074
**IL-6**	0.02	-0.03, 0.07	0.4
**IL-8**	0.14	-0.07, 0.34	0.2
**IL-10**	-0.13	-0.26, 0.00	0.044*
**IL-1-RA**	-0.01	-0.01, -0.01	<0.001*
**P/F**	0.04	0.01, 0.08	0.024*
**ERFE**	0.88	-0.56, 2.3	0.2
**HEPC**	-0.04	-0.07, 0.00	0.060
**STFR**	-0.01	-0.01, 0.00	0.005*
**TF**	-3.6	-9.7, 2.5	0.2
**CCI**	-1.2	-4.6, 2.2	0.5

Table S13- Multivariate Linear Regression Analysis. Dependent variable: hepcidin. Independent variables: age, gender, Hb, RDW, FT, N/L ratio, ERFE, sTfR IL10, IL6, IL8, IL1-RA, hepcidin, transferrin, P/F ratio and CCI=Charlson Comorbidity Index. Std. Beta = standardized regression coefficient, compares the relative importance of predictors. CI=Confidence Interval. P value indicates whether the predictor is statistically significant. "*" indicates statistically significant p values. RDW= Red Blood Cell Distribution Width; HB=Hemoglobin; FT= Ferritin; N/L ratio= Neutrophils over Lymphocytes ratio; ERFE=erythroferrone; sTfR= Soluble Transferrin Receptor; P/F ratio= ratio of arterial Oxygen Partial Pressure to Fractional inspired Oxygen; Tf=Transferrin.

	STD. BETA	95% CI	P VALUE
**AGE**	-0.09	-1.9, 1.7	>0.9
**GENDER [M]**	13	-17, 42	0.4
**RDW**	-6.9	-16, 1.9	0.12
**HB**	7.6	-1.1, 16	0.085
**FT**	0.04	0.03, 0.06	<0.001*
**N/L**	3.0	1.2, 4.8	<0.001*
**IL-6**	0.33	0.13, 0.54	0.001*
**IL-8**	-0.84	-1.7, -0.01	0.049*
**IL-10**	-0.07	-0.60, 0.46	0.8
**IL-1-RA**	0.03	0.01, 0.04	<0.001*
**P/F**	0.13	-0.02, 0.28	0.083
**ERFE**	-3.7	-9.5, 2.2	0.2
**IRON**	-0.59	-1.2, 0.03	0.060
**STFR**	0.00	-0.02, 0.01	0.9
**TF**	-28	-52, -3.0	0.028*
**CCI**	-0.99	-15, 13	0.9

Table S14-Logistic regression model for COVID-19 severity. Dependent variable: severity. Independent variables: age, gender, ferritin, N/L ratio, IL-10, IL-6, IL-8, RDW, CRP, P/F, hepcidin, therapy, CCI, heparin therapy, steroid therapy, antivirals therapy. Beta=unstandardized regression coefficient, indicates the magnitude of effect. Exp(B)= Odds Ratio, if Exp(B) > 1, the odds of the outcome increase as the predictor increases. P value indicates whether the predictor is statistically significant. "*" indicates statistically significant p values. P/F ratio= ratio of arterial Oxygen Partial Pressure to Fractional inspired Oxygen; RDW= Red Blood Cell Distribution Width; N/L= Neutrophils over Lymphocytes ratio; CRP= C-reactive protein; FT= Ferritin, CCI= Charlson Comorbidity Index. °= the lack of variability and the insufficient size of the comparison group prevented the model from reliably calculating the Odds Ratio and the p-value of steroid therapy.

	BETA (B)	OR (EXP(B))	P VALUE
**AGE**	0.00	1.00	0.9
**GENDER(M)**	-0.52	0.59	0.2
**RDW**	-0.08	0.92	0.6
**FT**	0.00	1.00	0.3
**HEPCIDIN**	0.01	1.01	0.004*
**IL-10**	0.00	1.00	0.6
**IL-6**	0.01	1.01	0.042*
**IL-8**	0.01	1.01	0.057
**sTfR**	0.00	1.00	0.022*
**N/L**	0.05	1.05	0.086
**CRP**	-0.01	0.99	<0.001*
**P/F**	-0.02	0.98	<0.001*
**CCI**	0.29	1.34	0.13
**HEPARIN THERAPY**	0.32	1.38	0.6
**STEROID THERAPY°**			
**ANTIVIRAL THERAPY**	0.10	1.10	0.8
